# Characterization of carbon, nitrogen, and phosphorus stoichiometry of plant leaves in the riparian zone of Dahuofang Reservoir

**DOI:** 10.1002/ece3.70152

**Published:** 2024-08-09

**Authors:** Baoliang Chang, Wei Chen, Xingyuan He, Shuai Yu

**Affiliations:** ^1^ CAS Key Laboratory of Forest Ecology and Silviculture Institute of Applied Ecology, Chinese Academy of Sciences Shenyang China; ^2^ Liaoning Shenyang Urban Ecosystem National Observation and Research Station Shenyang China; ^3^ Shenyang Arboretum, Chinese Academy of Sciences Shenyang China; ^4^ University of Chinese Academy of Sciences Beijing China

**Keywords:** leaves, life type, riparian zone, stoichiometric characteristics, woody plants

## Abstract

Carbon (C), nitrogen (N), and phosphorus (P) are essential nutrients that promote plant growth and development and maintain the stability of ecosystem structure and function. Analyzing the C, N, and P characteristics of plant leaves aids in understanding the plant's nutrient status and nutrient limitation. Seasonal water‐level fluctuations in riparian zones lead to various ecological problems, such as reduced biodiversity and decreased ecosystem stability. Therefore, comprehending the stoichiometric characteristics of riparian zone plants and their nutrient response to plant traits is important for a deeper insight into riparian zone forest ecosystems. This study analyzed the C, N, and P contents of the leaves of 44 woody plants in the riparian zone of the Dahuofang Reservoir to investigate the stoichiometric characteristics of C, N, and P of trees in the region. The results showed that the average C content of the leaves in woody plants was 446.9 g kg^−1^; the average N content was 28.42 g kg^−1^; and the average P content was 2.26 g kg^−1^. Compared to global and regional scales, woody plants in the riparian zone of the Dahuofang Reservoir exhibited higher N and P contents but lower N:P ratios. Compared to other riparian zones, woody plant leaves in the riparian zone of Dahuofang Reservoir had relatively high N content and N:P ratios. Variations in plant stoichiometric characteristics across different life forms were minimal, with only tree leaf P content significantly lower than its in shrubs. There was no significant correlation between leaf C, N, and P in woody plants, while specific leaf area showed a negative correlation with leaf C content. Trees in the riparian zone have high leaf N and P content and are primarily N‐limited during the growing season. This study reveals the stoichiometric characteristics of leaves of woody plants in the riparian zone, which can contribute to an in‐depth understanding of leaf stoichiometric patterns and the factors influencing them among plant life types in the riparian zone.

## Graphical abstract

Exploring the stoichiometric characteristics of plant leaves in riparian zones. Exploring the relationship between specific leaf area and C, N, P.

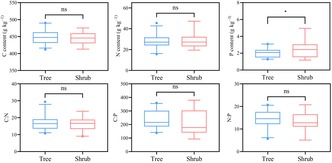

## 1 INTRODUCTION

Riparian zones are regions where terrestrial and aquatic ecosystems intertwine due to the seasonal fluctuations in water levels (Yang et al., 2021). Trees in riparian zones play an important role in ecological functions for both aquatic and wetland ecosystems (Dunea et al., 2021). Riparian trees contribute significantly to improving the resistance of riverbanks against soil erosion and provide multiple ecological functions, including pollutant filtration, biodiversity protection, and hydrological regulation (Capobianco et al., 2021; Hubble et al., 2010). In other words, a stabilized riparian forest ecosystems act as a buffer within the aquatic ecosystems in response to abiotic variability (Lozanovska et al., 2018). However, cyclical water‐level fluctuations and human activities have led to issues such as vegetation degradation and biodiversity decline.

Ecological stoichiometry is the science of studying the equilibrium relationships between multiple chemical elements in ecological processes (Elser et al., 2000; Güsewell, 2004). For plants, analyzing the relationships between elements can reflect their nutrient utilization capacity and ecosystem stability. Plant growth depends not only on the availability of individual nutrient but also on the balance among them (Han et al., 2013; He et al., 2020). In most cases, stoichiometric homeostasis is positively correlated with vegetation stability and functioning (Yu et al., 2010). Carbon (C), nitrogen (N), and phosphorus (P) play crucial roles in plant growth and community construction (Ågren, 2008). C is the basic element in plant composition, which can form organic matter and plays an important role in providing energy and building cellular structures for plants. N is a vital nutrient element in the plants, which convert nitrogen into biomolecules such as amino acids, proteins, and nuclei for cell growth and development. P is involved in regulating physiological processes such as energy conversion, photosynthesis, and cell division, and it is a constituent of important plant compounds. C, N, and P, as essential elements in plant growth, whose biochemical functions are coupled in the plant (Achat et al., 2016). C:N and C:P indicate plant nutrient utilization efficiency and growth rates, while N:P has been used to evaluate nutrient limitation and nitrogen saturation in ecosystems (Tessier & Raynal, 2003). Ecological stoichiometry investigates ecological processes and roles through homeostatic relationships among these elements, providing insight into understanding survival strategies developed by plants adapting to environmental changes.

In recent years, the pattern of plant stoichiometry across diverse study regions or scales has been extensively investigated. These investigations have elucidated the interrelationships among elements in different plant organs and the impacts of external factors such as altitude, geographic distribution, and water conditions on plant growth and development (Reich & Oleksyn, 2004). Riparian plants adjust elemental content in their bodies to adapt the changes in the environment, including periodic soil saturation and changes in soil chemistry (Pezeshki, 2001). Therefore, the study of plant stoichiometric characteristics will also be able to reflect the overall ecological situation of plants in riparian zones (Modrak et al., 2017). It has been shown that more active organs have a higher ability to maintain relatively stable elemental contents and ratios, showing a pattern of leaves > stems > roots in plants (Zhang, Wang, et al., 2018; Zhang, Zhao, et al., 2018). Therefore, nutrient stoichiometric characteristics of leaves are often indicative of plant‐specific responses to environmental factors in riparian zones (Chen et al., 2020). For example, the stoichiometric characteristics of plant leaves can be used as an indicator of plant response to flooding (González et al., 2010) and eutrophication (Huang et al., 2019).

Plants frequently adapt to the external environment by adjusting nutrient content in the leaves, thereby influencing various activities including photosynthesis, which correlates with leaf morphological traits (de la Riva et al., 2016; Wang et al., 2024; Zhang et al., 2017). Among the various leaf morphological traits, specific leaf area (SLA) stands out as an important index to characterizing plant leaf morphology (An & Shangguan, 2010; Osnas et al., 2013). Plants tend to show certain regular changes in leaf elemental content and SLA in order to acclimate environmental changes (Zhang, Wang, et al., 2018). It has been shown that SLA is significantly correlated with leaf N content in broadleaf species in southeastern China (Wu et al., 2012), and a general trend of high SLA correlating with lower leaf C content in non‐graminaceous angiosperms worldwide (Xing et al., 2021).

Afforestation plays an important role in water conservation, soil erosion prevention, and ecological improvement. However, nutrient balances in riparian vegetation restoration are frequently overlooked, with limited attention given to changes in the stoichiometric characteristics of riparian vegetation (Ding et al., 2022; Jing et al., 2022). In this study, we collected stoichiometric data of tree leaves from global and Chinese regions from published studies and compared them with this study. Our main purposes are (1) characterizing the carbon, nitrogen, and phosphorus content and stoichiometric characteristics of tree leaves in riparian zones. (2) Exploring the impact of differences in lifestyle and leaf morphology on leaf C, N, and P content. This study can reveal the difference pattern of ecological stoichiometric characteristics of vegetation around mountain reservoirs in cold regions and provide data support for plant screening in the process of vegetation restoration around reservoirs.

## 2 MATERIALS AND METHODS

### 2.1 Study area and species data

**FIGURE 1:**
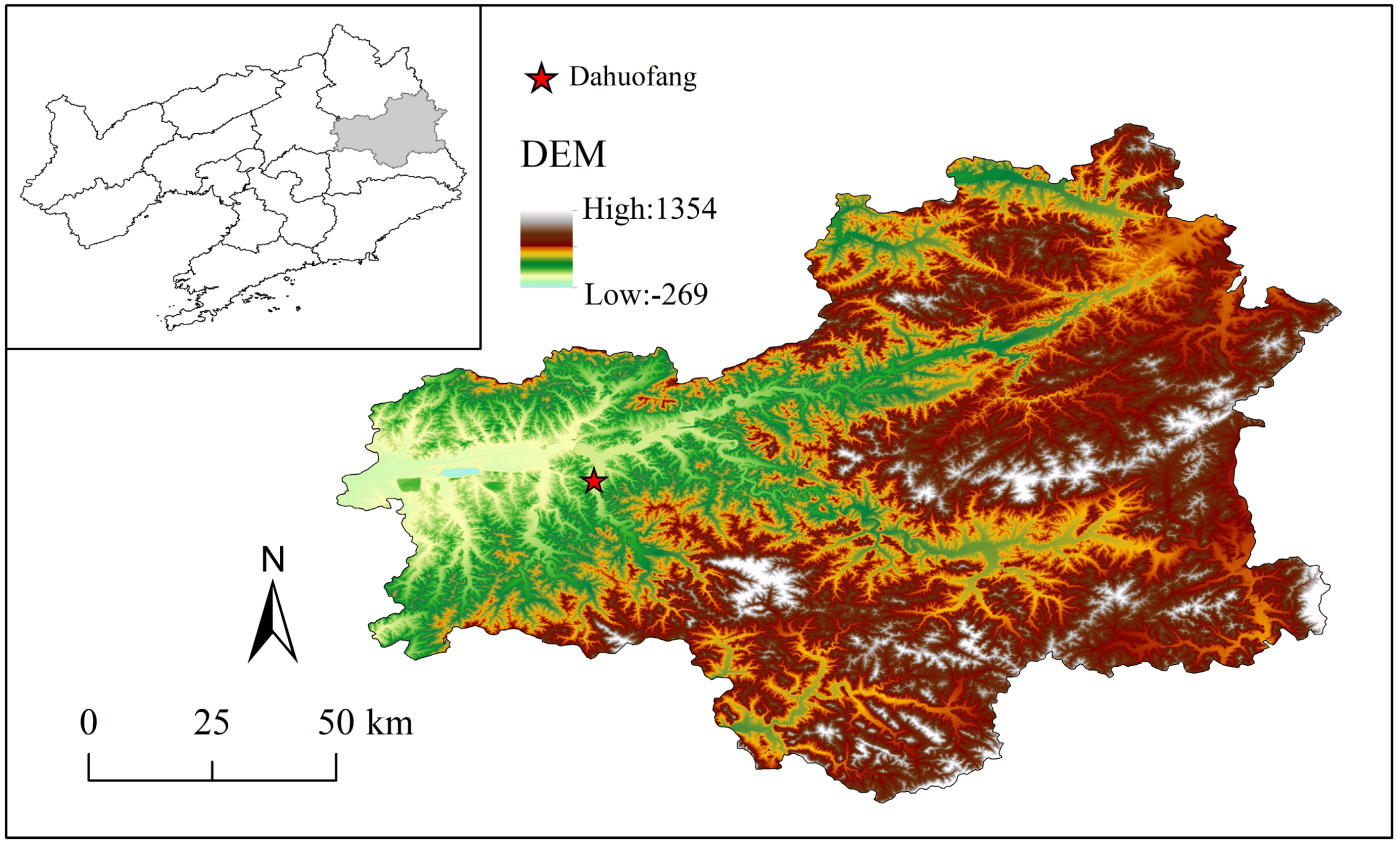
Study area.

Dahuofang Reservoir (E: 124°04′–124°21′, N: 41°50′–41°56′) is located in Fushun City, Liaoning Province. Located in the middle reaches of the Hun River in the eastern suburb of Fushun City, the reservoir is narrow and long, about 35 km in length from east to west, and is one of the most important water sources for the cities of Shenyang and Fushun. The reservoir area is densely wooded in the north and south, which is a typical riverbank area. Dahuofang Reservoir has a typical temperate continental semihumid climate with an average annual temperature of 5–8°C. The average annual precipitation is about 800–1000 mm, with a total reservoir capacity of 2.268 billion cubic meters, and the region is dominated by oil pine plantation forests, larch plantation forests, and acacia natural secondary forests, and the soil type is mainly brown loam (Yan et al., 2016). We selected 44 common woody plants in the riparian zone, and the detailed list is shown in Table 1 and Figure 1.

**TABLE 1:** List of 44 woody plants in the riparian zone of the Dahuofang Reservoir.

No.	Family	Tree	No.	Family	Shrub
1	Araliaceae	*Aralia elata*	1	Actinidiaceae	*Actinidia arguta*
2	Betulaceae	*Betula costata*	2	Araliaceae	*Eleutherococcus sessiliflorus*
3	Fabaceae	*Gleditsia microphylla*	3	Betulaceae	*Corylus heterophylla*
4		*Robinia pseudoacacia*	4	Caprifoliaceae	*Lonicera maackii*
5	Fagaceae	*Quercus mongolica*	5		*Lonicera chrysantha*
6	Juglandaceae	*Pterocarya stenoptera*	6	Celastraceae	*Euonymus maackii*
7		*Juglans mandshurica*	7		*Celastrus flagellaris*
8	Malvaceae	*Tilia mandshurica*	8		*Euonymus alatus*
9	Moraceae	*Morus alba*	9	Fabaceae	*Amorpha fruticosa*
10	Oleaceae	*Fraxinus mandshurica*	10		*Lespedeza bicolor*
11		*Syringa reticulata*	11	Hydrangeaceae	*Philadelphus pekinensis*
12		*Fraxinus chinensis*	12	Phyllanthaceae	*Flueggea suffruticosa*
13	Rosaceae	*Prunus padus*	13	Rhamnaceae	*Rhamnus ussuriensis*
14		*Malus baccata*	14	Rosaceae	*Rosa davurica*
15		*Prunus mandshurica*	15		*Prunus tomentosa*
16		*Crataegus pinnatifida*	16	Salicaceae	*Salix integra*
17	Rutaceae	*Phellodendron amurense*	17		*Salix schwerinii*
18	Salicaceae	*Sali matsudana*	18	Sapindaceae	*Acer tataricum*
19		*Salix koreensis*	19	Viburnaceae	*Sambucus williamsii*
20		*Populus alba*			
21	Sapindaceae	*Acer saccharum*			
22		*Acer pictum*			
23		*Acer truncatum*			
24	Ulmaceae	*Ulmus pumila*			
25		*Ulmus davidiana*			

### 2.2 Sampling and measurement

During the plant growing season (August 2016), we selected 44 representative woody plants from the riparian zone around the Dahuofang Reservoir through field trekking (Table 1). For each tree species, three well‐lived and uniform plants were selected, and well‐grown and mature plant leaves were collected in each of the four directions of the crown, east, west, south, north, and south. Each tree species had 12 leaves, and the leaves were brought back to the laboratory and rinsed with deionized water. Leaf area was calculated by taking photographs of leaf surfaces with a digital camera. The oven was used to blanch the leaves at 105°C for 30 min, dried at 85°C to constant weight, weighed for dry matter mass, and then the samples were pulverized to be measured. Leaf C and N were measured by an elemental analyzer (Elementar Vario MAX, Germany); P was determined by the molybdenum‐antimony antimony colorimetric method, and the measurements were repeated three times for each sample. SLA is the ratio of leaf area to dry matter mass.

Three sampling points were selected in the plant leaf sampling area, and three replicate soil samples were taken from each sampling point and brought back to the laboratory in quadrature for testing. Soil moisture content was determined using a soil moisture tachometer (TDR200); pH was determined by potentiometric method; soil organic carbon content was determined by potassium dichromate plus thermal oxidation method; total nitrogen (TN) content was determined by Kjeldahl method; and ammonium nitrogen (NH^4+^–N) was determined using a continuous flow analyzer (Autoanalyzer).

### 2.3 Data analysis

We used one‐way ANOVA to compare the stoichiometric characteristics of trees and shrubs (*p* < .05). Pearson correlation analysis was used to investigate the relationship between leaf C, N, P, and stoichiometric ratios of each factor through the R package “corrplot”; the relationship between leaf C, N, P, and other factors was analyzed through the linear fitting. GraphPad 9.1 was used for plotting.

## 3 RESULTS

### 3.1 Species composition, soil parameter, and leaf stoichiometry

The 44 species of woody plants in the riparian zone of Dahuofang Reservoir belong to 20 families and 32 genera. Of these, Rosaceae, Populusaceae, Sapotaceae, and Leguminosae accounted for 43% of the total. It can be categorized by life type into 25 species of trees and 19 species of shrubs. The coefficient variations (CVs) of soil parameters in this study ranged from 0.06 to 0.48. The soil showed some acidity with a pH of 6.08. The soil SOC and TN concentrations were 58.76 and 4.1 g kg^−1^ respectively. the NH^4+^–N content was 14.24 mg kg^−1^. the soil water content was 61.12% (Table 2).

**TABLE 2:** General information on soil parameters in the riparian zone of the Dahuofang Reservoir.

Item	SOC (g kg^−1^)	TN (g kg^−1^)	${\mathrm{NH}}_{4}^{+}$–N (mg kg^−1^)	C:N	pH	Soil water content%
Mean	58.76	4.10	14.24	13.94	6.08	61.12
CV	0.48	0.36	0.38	0.13	0.06	0.29

Abbreviation: CV, coefficient variations.

There was moderate variability in leaf C, N, and P contents and their ratios for 44 woody plants in the riparian zone of Dahuofang Reservoir (Table 3). Among them, the CV of C content is the smallest, with a value of 0.04. The range of variation of leaf C content was 412.06–490.43 g kg^−1^. The mean value was 446.9 g kg^−1^. The CV for leaf N content was 0.24. Leaf N content ranged from 15.64–47.20 g kg^−1^ with a mean value of 28.42 g kg^−1^. The largest CV was leaf P content, with a value of 0.38. Leaf P content ranged from 1.18 to 4.95 g kg^−1^ with a mean value of 2.26 g kg^−1^. The mean values of the stoichiometric ratios C:N, C:P, and N:P for the three elements were 16.64, 224.03, and 13.8, respectively. The leaves of the present study plants had higher N content and P content as compared to the leaves of global woody plants and Chinese plants. However, leaf N:P of woody plants in this study was lower than that of global or all China plants and higher than that of wetland plants in China (Table 4). The average leaf C concentration of trees in the riparian zone was higher than that of Chinese regions and Chinese subtropical wetlands, leaf N concentration was higher than that of the global and other Chinese regions, and leaf P concentration was at the same level as the values of plants in Chinese wetlands and Chinese subtropical wetlands.

**TABLE 3:** General characteristics of leaf C, N, and P stoichiometry of 44 plant species (g kg^−1^).

Variable	Mean	Median	Maximum	Minimum	CV
C	446.9	446.54	490.43	412.06	0.04
N	28.42	27.20	47.20	15.64	0.24
P	2.26	2.14	4.95	1.18	0.38
C:N	16.64	16.54	29.39	9.09	0.25
C:P	224.03	207.83	379.25	92.92	0.35
N:P	13.8	13.80	20.75	5.11	0.30

Abbreviation: CV, coefficient variations.

**TABLE 4:** Reported leaf stoichiometric characteristics of woody plants.

Study area	C (g kg^−1^)	N (g kg^−1^)	P (g kg^−1^)	N:P	C:N:P	Reference
Global woody plants		18.22	1.10	16.56		Tian et al. (2018)
Terrestrial ecosystems in China					427:19:1	Zhang et al. (2021)
Clusters in China	436.8	14.14	1.11	12.74	394:13:1	Tang et al. (2018)
Forests in China		16.36	1.32	14.12		Liu et al. (2019)
Woody plants in China		20.77	1.58	17.28		Duan (2023)
Wetlands of China		18.3	2.55	7.18		Hu et al. (2021)
Subtropical Wetlands of China	422.14	22.33	2.25	9.92	188:10:1	Yu et al. (2020)
Dahuofang Reservoir riparian zone	446.9	28.42	2.26	12.57	198:13:1	This study

### 3.2 Leaf stoichiometry of different life types

**FIGURE 2:**
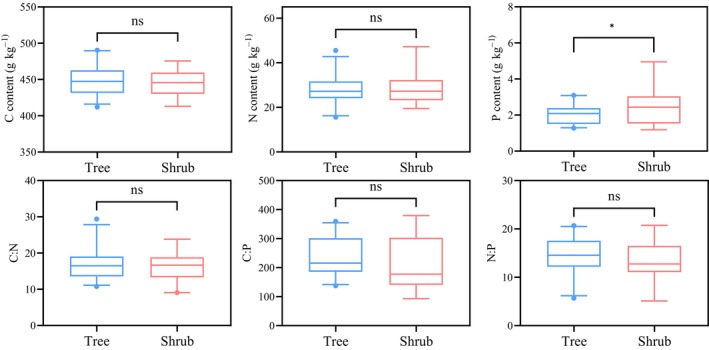
Comparison of plant leaf stoichiometric characteristics between trees and shrubs.

Most of the C, N, and P contents and their stoichiometric ratios were not significant between the leaves of different life types of plants (Figure 2). The differences in C and N contents between 25 trees and 19 shrubs were not significant (*p* > .05), but there was a significant difference in P contents, with shrubs having higher P contents than trees (*p* < .05). The average contents of C, N, and P of 25 tree species were 447.81, 28.07, and 2.03 g kg^−1^, respectively. The average contents of C, N, and P of 19 shrub species were 445.71, 28.88, and 2.56 g kg^−1^, respectively. The ratio between C, N, and P showed that both tree C:P and N:P were higher for trees than shrubs, but did not reach the significant level (*p* > .05).

### 3.3 Relationship between C, N, P content and its stoichiometric ratio

**FIGURE 3:**
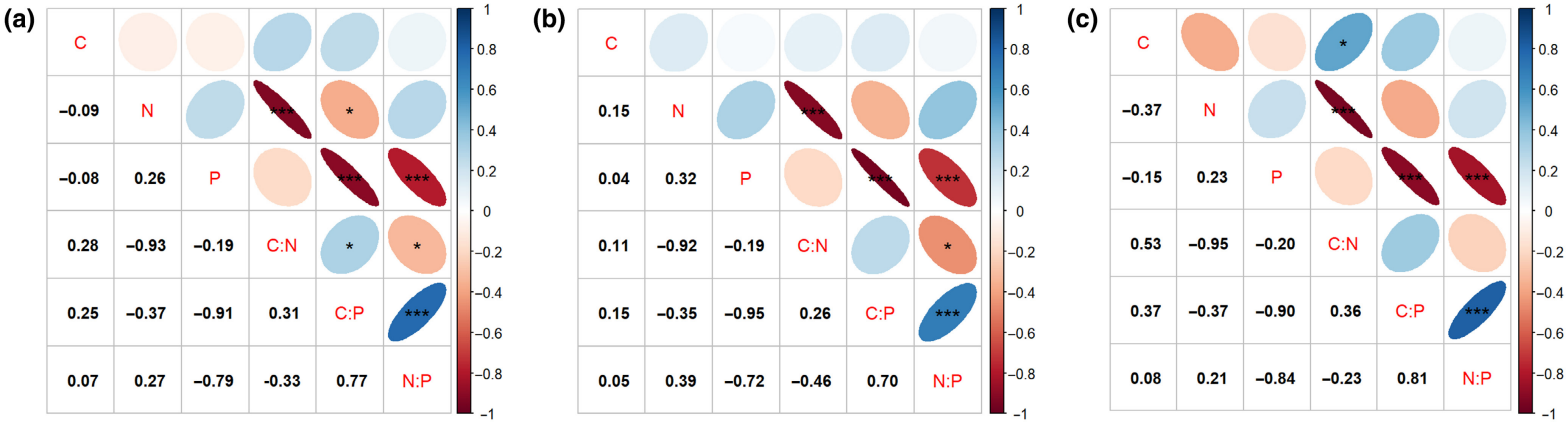
Relationship between C, N, P and their stoichiometric ratios (a) All plants (b) Trees (c) Shrubs.

This study correlated leaf C, N, P and their ecological stoichiometric characteristics of woody plants in the riparian zone (Figure 3). By exploring the relationship between plant leaf C, N, P and their stoichiometric ratios through correlation analysis, we found that there was no significant correlation between the leaf C, N, and P elemental contents of woody plants in riparian areas. There was a significant negative correlation between N and C:N, N, and C:P; P showed a highly significant negative correlation with C:P and N:P. Our analysis of woody plants of different life types revealed that leaf C content of shrubs showed a significant positive correlation with C:N. Significant positive correlations of C:N and C:P in all woody plants turned out to be non‐significant in both trees and shrubs.

### 3.4 Relationship between elemental content of plant leaves and SLA

**FIGURE 4:**
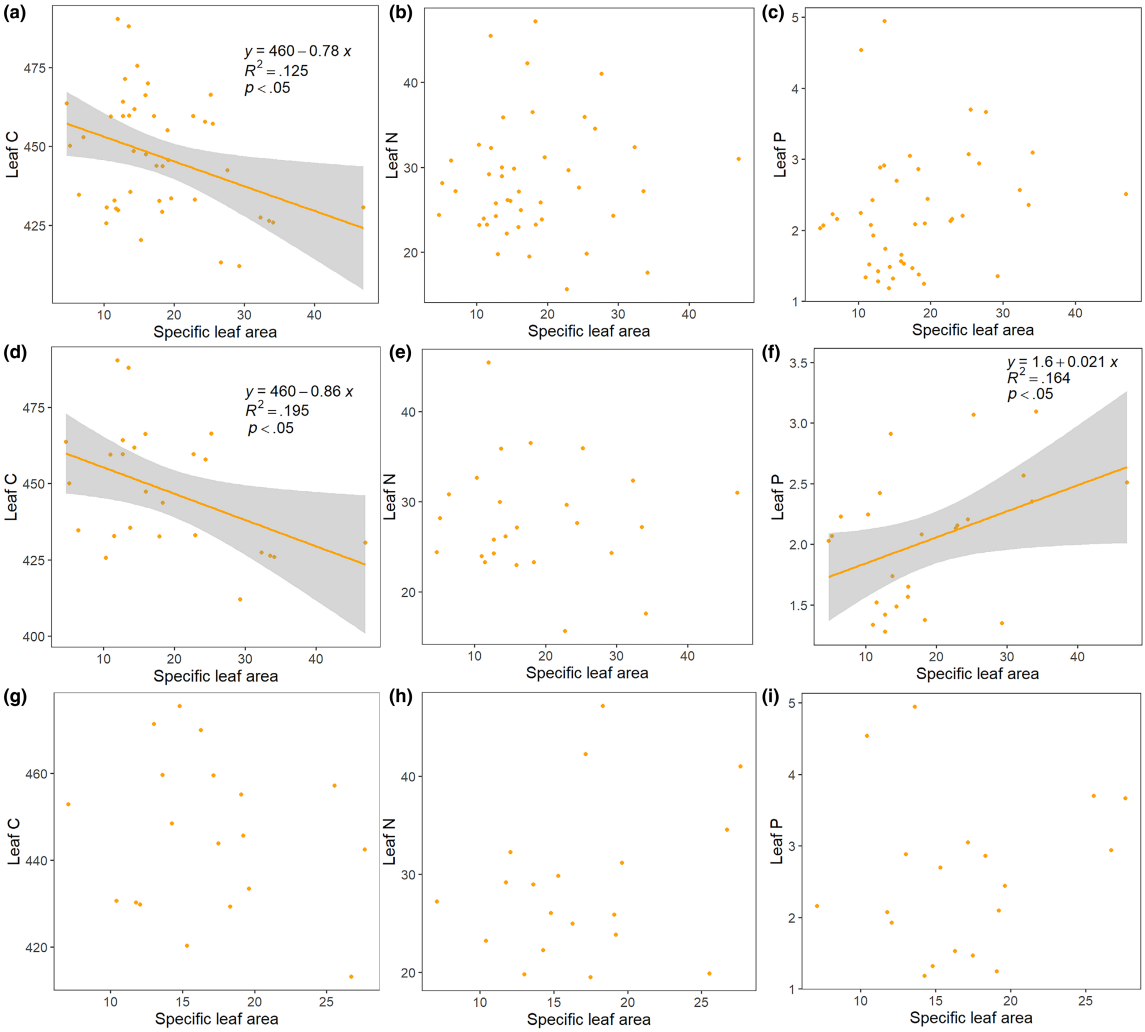
Relationship between leaf C, N, P content and SLA. (a–c) all plants (d–f) trees (g–i) shrubs.

As can be seen from Figure 4, the SLA of woody plants in the riparian zone of Dahuofang Reservoir was not significantly correlated with N and P content (*p* > .05) and showed a significant negative correlation with C content. In trees, SLA also showed a significant positive correlation with P content. In shrubs, none of the plant leaf SLA showed significant correlation (*p* > .05) with elemental content.

## 4 DISCUSSION

### 4.1 Characterization of leaf ecological stoichiometry of plants in riparian zones

This study showed that the average N and P contents of woody plant leaves in the study area were significantly higher than the average contents of Chinese plant leaves, and even the average N contents were higher than the average contents of plant leaves in other wetland areas (Table 3). In addition to this, the differences in plant leaf C and N contents were not significant between life types, but P contents were significantly lower in trees than in shrubs. This may be due to the fact that plant foliar nutrient concentration increases with decreasing plant size, especially P element (Elser et al., 2010). Plant C content in riparian zones is higher than the national average, probably because plants have adapted to the environment by increasing the C content of plant tissues to ensure normal growth (Rong et al., 2015). Leaf N content of woody plants in the riparian zone was significantly higher than the national average, which may be related to the driving mechanism of leaf N by the wet environment. Because plants allocate more N in nonsoluble protein fibers to adapt to the environment, they have higher specific leaf weights and thus tend to have higher leaf N contents (Pan et al., 2020; Reich et al., 1998). The P content of leaves reflects both the P supply capacity of the soil and the efficiency of P uptake and utilization by plants. The P content of plant leaves in this study was significantly higher than the average of the other regions and was approximately the same as the P content of plant leaves in other wetland regions (Table 3).

To a certain extent, leaf elemental stoichiometric characteristics can reflect the resource acquisition strategies of plants and can clarify the nutrient limitations of plant growth (Ågren et al., 2012; Townsend et al., 2007). Plant leaf C:N and C:P reflect the efficiency of N and P utilization by the plant, and N:P reflects the relative limitation of the plant by N or P (Hessen et al., 2007). Larger C:N and C:P values indicate more efficient utilization of N and P elements by plants. In the present study, the C content of woody plants in the riparian zone was higher and the degree of variability was less. Leaf C:N and C:P values were lower than national averages, indicating that woody plants in this study were less efficient in utilizing N and P. The leaf C:N and C:P values were lower than national averages. Meanwhile, leaf N content was negatively correlated with C:N, leaf P with N:P, and P with C:P (*p* < .01), indicating that the efficiency of N and P utilization decreased with increasing N and P content within a certain range, which was consistent with the results of the previous study (Rong et al., 2015). For N:P, terrestrial plant ecosystems and wetland ecosystems have different limits. Plants tend to be N‐limited for terrestrial plants with N:P < 10 and P‐limited for terrestrial plants with N:P > 20 (Güsewell, 2004). In wetland ecosystems, plant growth is N‐limited when plant leaf N:P < 14 and mainly P‐limited when plant leaf N:P > 16 (Koerselman & Meuleman, 1996). It has been shown that plant growing seasons in riparian zones are usually limited by N (Rong et al., 2015). In the present study, the mean and median leaf nitrogen–phosphorus ratio of 44 woody plants was 13.8, indicating that most of the woody plants in the study area were mainly N‐limited during the growing season. This may be due to the high soil water content and the solubility of soil effective phosphorus in water, resulting in the leaves of plants in the riparian zone having higher P content (Table 2) (Li et al., 2020; Shu et al., 2017). This also indicates that riparian zones have unique characteristics compared to other regions, as riparian zones with sufficient soil moisture can enable soil nitrogen and phosphorus to converge and improve plant absorption efficiency.

The magnitude of variation in leaf stoichiometries varies among plants, reflecting the internal stability of plant leaf C, N, and P contents and their different sensitivities to changes in the ecological environment (Tao et al., 2016). In this study, the CV of leaf C content of 44 woody plants in the riparian zone was the smallest, while the CV of N and P content was larger. This is mainly because carbon is the most important element of plant dry matter, accounting for about half of plant biomass and playing a decisive role in plant growth, and N and P are limiting nutrients necessary for plant growth (Tao et al., 2016), and their contents vary with the developmental stage of the plant body, which is in line with the results of previous ecological stoichiometric characterization studies (Liu et al., 2020; Rong et al., 2015).

### 4.2 Relationship between leaf stoichiometry and SLA

Plants adopt a range of adaptive strategies in the face of environmental change, and such adaptive strategies can optimize their photosynthesis, survival, and defense functions by adjusting leaf morphological traits and stoichiometric ratios (Castellanos et al., 2018). It has been shown that plants in frequently flooded areas tend to have higher SLA and plants in less frequently flooded areas tend to have lower SLA (McCoy‐Sulentic et al., 2017). Thus, the stoichiometry of plant leaves is closely related to the morphology of the leaves, and the elemental concentrations of the leaves tend to vary according to SLA (Li et al., 2021; Wu et al., 2012). In this study, there was a negative correlation between SLA and C in the leaves of 44 woody plants in the riparian zone, which is consistent with previous reports (Xing et al., 2021). Therefore, it can be inferred that the larger SLAs of woody plants in most riparian zones may result in plants with lower C, but this conclusion may not apply to all plants and remains to be verified in the context of more herbaceous plants. In trees, plant leaf P content showed a significant positive correlation with SLA, suggesting that in trees, a larger SLA implies that plant leaves have a higher P content. The correlations between SLA and elements C, N, and P in this study suggest that the relationship between plant leaf traits and nutrient elements may be related to the functions that the elements exercise in the plant, and that there are differences between plants of different life types (Minden & Kleyer, 2014; Yan et al., 2015).

## 5 CONCLUSION

In this study, we investigated the nutrient stoichiometric characteristics of leaves of woody plants in the riparian zone, comparing differences between life types and relating them to morphological indicators of leaves. Our results showed that leaf N and P contents were higher than the global average, and leaf N contents were higher than the levels in other wetland areas, but leaf N:P was higher than the levels in other wetland areas. Differences in C and N content between trees and shrubs were not significant, but trees had lower P content compared to shrubs. Characteristics were relatively uniform among trees and varied considerably among shrubs. SLA showed a negative correlation with leaf C content. Our results confirm the regional characteristic differences in leaf stoichiometry of vegetation in cold riparian zones, and the results may provide important information to support effective vegetation restoration and sustainable management in cold riparian zones.

## AUTHOR CONTRIBUTIONS

**Baoliang Chang:** Conceptualization (equal); data curation (equal); methodology (equal); writing – original draft (equal). **Xingyuan He:** Conceptualization (equal); project administration (equal); supervision (equal); visualization (equal). **Wei Chen:** Project administration (equal); supervision (equal); visualization (equal). **Shuai Yu:** Conceptualization (equal); funding acquisition (equal); supervision (equal); visualization (equal); writing – review and editing (equal).

## FUNDING INFORMATION

This research was funded by The Youth Innovation Promotion Association CAS, grant number 2022195 and Natural Science Foundation of Liaoning Province of China (2024‐MSBA‐89).

## CONFLICT OF INTEREST STATEMENT

The authors declare that they have no known competing financial interests or personal relationships that could have appeared to influence the work reported in this paper.

## Data Availability

The complete manuscript data can be accessed at Supporting Information.

## References

[ece370152-bib-0001] Achat, D. L. , Augusto, L. , Gallet‐Budynek, A. , & Loustau, D. (2016). Future challenges in coupled C‐N‐P cycle models for terrestrial ecosystems under global change: A review. Biogeochemistry, 131(1–2), 173–202. 10.1007/s10533-016-0274-9

[ece370152-bib-0002] Ågren, G. I. (2008). Stoichiometry and nutrition of plant growth in natural communities. Annual Review of Ecology, Evolution, and Systematics, 39, 153–170. 10.1146/annurev.ecolsys.39.110707.173515

[ece370152-bib-0003] Ågren, G. I. , Wetterstedt, J. , & Billberger, M. F. K. (2012). Nutrient limitation on terrestrial plant growth ‐ modeling the interaction between nitrogen and phosphorus. New Phytologist, 194(4), 953–960. 10.1111/j.1469-8137.2012.04116.x 22458659

[ece370152-bib-0004] An, H. , & Shangguan, Z. P. (2010). Leaf stoichiometric trait and specific leaf area of dominant species in the secondary succession of the loess plateau. Polish Journal of Ecology, 58(1), 103–113.

[ece370152-bib-0005] Capobianco, V. , Robinson, K. , Kalsnes, B. , Ekeheien, C. , & Hoydal, O. (2021). Hydro‐mechanical effects of several riparian vegetation combinations on the streambank stability–a benchmark case in southeastern Norway. Sustainability, 13(7), 4046. 10.3390/su13074046

[ece370152-bib-0006] Castellanos, A. E. , Llano‐Sotelo, J. M. , Machado‐Encinas, L. I. , López‐Piña, J. E. , Romo‐Leon, J. R. , Sardans, J. , & Peñuelas, J. (2018). Foliar C, N, and P stoichiometry characterize successful plant ecological strategies in the Sonoran Desert. Plant Ecology, 219(7), 775–788. 10.1007/s11258-018-0833-3

[ece370152-bib-0007] Chen, Y. S. , Stagg, C. L. , Cai, Y. J. , Lü, X. T. , Wang, X. L. , Shen, R. C. , & Lan, Z. C. (2020). Scaling responses of leaf nutrient stoichiometry to the lakeshore flooding duration gradient across different organizational levels. Science of the Total Environment, 740, 139740. 10.1016/j.scitotenv.2020.139740 32927530

[ece370152-bib-0008] de la Riva, E. G. , Olmo, M. , Poorter, H. , Ubera, J. L. , & Villar, R. (2016). Leaf mass per area (LMA) and its relationship with leaf structure and anatomy in 34 Mediterranean Woody species along a water availability gradient. PLoS One, 11(2), e0148788. 10.1371/journal.pone.0148788 26867213 PMC4750855

[ece370152-bib-0009] Ding, D. D. , Arif, M. , Liu, M. H. , Li, J. J. , Hu, X. , Geng, Q. W. , & Li, C. X. (2022). Plant‐soil interactions and C:N:P stoichiometric homeostasis of plant organs in riparian plantation. Frontiers in Plant Science, 13, 9023. 10.3389/fpls.2022.979023 PMC9376457 35979078

[ece370152-bib-0010] Duan, X. G. (2023). Stoichiometric characteristics of woody plant leaves and responses to climate and soil factors in China. PLoS One, 18(9), e0291957. 10.1371/journal.pone.0291957 37733819 PMC10513206

[ece370152-bib-0011] Dunea, D. , Bretcan, P. , Purcoi, L. , Tanislav, D. , Serban, G. , Neagoe, A. , & Iordache, S. (2021). Effects of riparian vegetation on evapotranspiration processes and water quality of small plain streams. Ecohydrology & Hydrobiology, 21(4), 629–640. 10.1016/j.ecohyd.2021.02.004

[ece370152-bib-0012] Elser, J. J. , Fagan, W. F. , Kerkhoff, A. J. , Swenson, N. G. , & Enquist, B. J. (2010). Biological stoichiometry of plant production: Metabolism, scaling and ecological response to global change. New Phytologist, 186(3), 593–608. 10.1111/j.1469-8137.2010.03214.x 20298486

[ece370152-bib-0013] Elser, J. J. , Sterner, R. W. , Gorokhova, E. , Fagan, W. F. , Markow, T. A. , Cotner, J. B. , Harrison, J. F. , Hobbie, S. E. , Odell, G. M. , Weider, L. W. , & Weider, L. J. (2000). Biological stoichiometry from genes to ecosystems. Ecology Letters, 3(6), 540–550. 10.1046/j.1461-0248.2000.00185.x

[ece370152-bib-0014] González, E. , Muller, E. , Comín, F. A. , & González‐Sanchis, M. (2010). Leaf nutrient concentration as an indicator of Populus and Tamarix response to flooding. Perspectives in Plant Ecology, Evolution and Systematics, 12(4), 257–266. 10.1016/j.ppees.2010.07.001

[ece370152-bib-0015] Güsewell, S. (2004). N:P ratios in terrestrial plants:: Variation and functional significance. New Phytologist, 164(2), 243–266. 10.1111/j.1469-8137.2004.01192.x 33873556

[ece370152-bib-0016] Han, W. X. , Tang, L. Y. , Chen, Y. H. , & Fang, J. Y. (2013). Relationship between the relative limitation and resorption efficiency of nitrogen vs phosphorus in woody plants. PLoS One, 8(12), e83366. 10.1371/journal.pone.0083366 24376694 PMC3871644

[ece370152-bib-0017] He, M. S. , Yan, Z. B. , Cui, X. Q. , Gong, Y. M. , Li, K. H. , & Han, W. X. (2020). Scaling the leaf nutrient resorption ef ficiency: Nitrogen vs phosphorus in global plants. Science of the Total Environment, 729, 138920. 10.1016/j.scitotenv.2020.138920 32371208

[ece370152-bib-0018] Hessen, D. O. , Jensen, T. C. , Kyle, M. , & Elser, J. J. (2007). RNA responses to N‐ and P‐limitation; reciprocal regulation of stoichiometry and growth rate in *Brachionus* . Functional Ecology, 21(5), 956–962. 10.1111/j.1365-2435.2007.01306.x

[ece370152-bib-0019] Hu, Y. K. , Liu, X. Y. , He, N. P. , Pan, X. , Long, S. Y. , Li, W. , Zhang, M. , & Cui, L. J. (2021). Global patterns in leaf stoichiometry across coastal wetlands. Global Ecology and Biogeography, 30(4), 852–869. 10.1111/geb.13254

[ece370152-bib-0020] Huang, D. , Wang, D. M. , & Ren, Y. (2019). Using leaf nutrient stoichiometry as an indicator of flood tolerance and eutrophication in the riparian zone of the Lijang River. Ecological Indicators, 98, 821–829. 10.1016/j.ecolind.2018.11.064

[ece370152-bib-0021] Hubble, T. C. T. , Docker, B. B. , & Rutherfurd, I. D. (2010). The role of riparian trees in maintaining riverbank stability: A review of Australian experience and practice. Ecological Engineering, 36(3), 292–304. 10.1016/j.ecoleng.2009.04.006

[ece370152-bib-0022] Jing, X. , Su, W. H. , Fan, S. H. , Luo, H. Y. , & Chu, H. Y. (2022). Ecological strategy of Phyllostachys heteroclada oliver in the riparian zone based on ecological stoichiometry. Frontiers in Plant Science, 13, 4124. 10.3389/fpls.2022.974124 PMC9659970 36388549

[ece370152-bib-0023] Koerselman, W. , & Meuleman, A. F. M. (1996). The vegetation N:P ratio: A new tool to detect the nature of nutrient limitation. Journal of Applied Ecology, 33(6), 1441–1450. 10.2307/2404783

[ece370152-bib-0024] Li, X. F. , Ding, C. X. , Bu, H. , Han, L. L. , Ma, P. , & Su, D. R. (2020). Effects of submergence frequency on soil C:N:P ecological stoichiometry in riparian zones of Hulunbuir steppe. Journal of Soils and Sediments, 20(3), 1480–1493. 10.1007/s11368-019-02533-x

[ece370152-bib-0025] Li, Y. Q. , He, W. , Wu, J. , Zhao, P. , Chen, T. , Zhu, L. W. , Ouyang, L. , Ni, G. , & Hölscher, D. (2021). Leaf stoichiometry is synergistically‐driven by climate, site, soil characteristics and phylogeny in karst areas, Southwest China. Biogeochemistry, 155(2), 283–301. 10.1007/s10533-021-00826-3

[ece370152-bib-0026] Liu, D. , Zhang, J. , Biswas, A. , Cao, J. J. , Xie, H. J. , & Qi, X. X. (2020). Seasonal dynamics of leaf stoichiometry of Phragmites australis: A case study from Yangguan wetland, Dunhuang, China. Plants‐Basel, 9(10), 101323. 10.3390/plants9101323 PMC7600640 33036307

[ece370152-bib-0027] Liu, J. X. , Fang, X. , Tang, X. L. , Wang, W. T. , Zhou, G. Y. , Xu, S. , Huang, W. , Wang, G. , Yan, J. , Ma, K. , Du, S. , Li, S. , Han, S. , & Ma, Y. X. (2019). Patterns and controlling factors of plant nitrogen and phosphorus stoichiometry across China's forests. Biogeochemistry, 143(2), 191–205. 10.1007/s10533-019-00556-7

[ece370152-bib-0028] Lozanovska, I. , Ferreira, M. T. , & Aguiar, F. C. (2018). Functional diversity assessment in riparian forests ‐ multiple approaches and trends: A review. Ecological Indicators, 95, 781–793. 10.1016/j.ecolind.2018.08.039

[ece370152-bib-0029] McCoy‐Sulentic, M. E. , Kolb, T. E. , Merritt, D. M. , Palmquist, E. , Ralston, B. E. , Sarr, D. A. , & Shafroth, P. B. (2017). Changes in community‐level riparian plant traits over inundation gradients, Colorado River, grand canyon. Wetlands, 37(4), 635–646. 10.1007/s13157-017-0895-3

[ece370152-bib-0030] Minden, V. , & Kleyer, M. (2014). Internal and external regulation of plant organ stoichiometry. Plant Biology, 16(5), 897–907. 10.1111/plb.12155 24552639

[ece370152-bib-0031] Modrak, P. , Brunzel, S. , & Lorenz, A. W. (2017). Riparian plant species preferences indicate diversification of site conditions after river restoration. Ecohydrology, 10(5), 1852. 10.1002/eco.1852

[ece370152-bib-0032] Osnas, J. L. D. , Lichstein, J. W. , Reich, P. B. , & Pacala, S. W. (2013). Global leaf trait relationships: Mass, area, and the leaf economics Spectrum. Science, 340(6133), 741–744. 10.1126/science.1231574 23539179

[ece370152-bib-0033] Pan, Y. J. , Cieraad, E. , Armstrong, J. , Armstrong, W. , Clarkson, B. R. , Colmer, T. D. , Pedersen, O. , Visser, E. J. W. , Voesenek, L. A. C. J. , & van Bodegom, P. M. (2020). Global patterns of the leaf economics spectrum in wetlands. Nature Communications, 11(1), 18354. 10.1038/s41467-020-18354-3 PMC7481225 32908150

[ece370152-bib-0034] Pezeshki, S. R. (2001). Wetland plant responses to soil flooding. Environmental and Experimental Botany, 46(3), 299–312. 10.1016/s0098-8472(01)00107-1

[ece370152-bib-0035] Reich, P. B. , & Oleksyn, J. (2004). Global patterns of plant leaf N and P in relation to temperature and latitude. Proceedings of the National Academy of Sciences of the United States of America, 101(30), 11001–11006. 10.1073/pnas.0403588101 15213326 PMC503733

[ece370152-bib-0036] Reich, P. B. , Walters, M. B. , Ellsworth, D. S. , Vose, J. M. , Volin, J. C. , Gresham, C. , & Bowman, W. D. (1998). Relationships of leaf dark respiration to leaf nitrogen, specific leaf area and leaf life‐span: A test across biomes and functional groups. Oecologia, 114(4), 471–482. 10.1007/s004420050471 28307896

[ece370152-bib-0037] Rong, Q. Q. , Liu, J. T. , Cai, Y. P. , Lu, Z. H. , Zhao, Z. Z. , Yue, W. C. , & Xia, J. B. (2015). Leaf carbon, nitrogen and phosphorus stoichiometry of Tamarix chinensis Lour. In the Laizhou Bay coastal wetland, China. Ecological Engineering, 76, 57–65. 10.1016/j.ecoleng.2014.03.002

[ece370152-bib-0038] Shu, X. , Zhang, K. R. , Zhang, Q. F. , & Wang, W. B. (2017). Response of soil physico‐chemical properties to restoration approaches and submergence in the water level fluctuation zone of the Danjiangkou reservoir, China. Ecotoxicology and Environmental Safety, 145, 119–125. 10.1016/j.ecoenv.2017.07.023 28728116

[ece370152-bib-0039] Tang, Z. Y. , Xu, W. T. , Zhou, G. Y. , Bai, Y. F. , Li, J. X. , Tang, X. L. , Chen, D. , Liu, Q. , Ma, W. , Xiong, G. , He, H. , He, N. , Guo, Y. , Guo, Q. , Zhu, J. , Han, W. , Hu, H. , Fang, J. , & Xie, Z. Q. (2018). Patterns of plant carbon, nitrogen, and phosphorus concentration in relation to productivity in China's terrestrial ecosystems. Proceedings of the National Academy of Sciences of the United States of America, 115(16), 4033–4038. 10.1073/pnas.1700295114 29666316 PMC5910803

[ece370152-bib-0040] Tao, Y. , Wu, G. L. , Zhang, Y. M. , & Zhou, X. B. (2016). Leaf N and P stoichiometry of 57 plant species in the Karamori Mountain ungulate nature reserve, Xinjiang, China. Journal of Arid Land, 8(6), 935–947. 10.1007/s40333-016-0019-6

[ece370152-bib-0041] Tessier, J. T. , & Raynal, D. J. (2003). Use of nitrogen to phosphorus ratios in plant tissue as an indicator of nutrient limitation and nitrogen saturation. Journal of Applied Ecology, 40(3), 523–534. 10.1046/j.1365-2664.2003.00820.x

[ece370152-bib-0042] Tian, D. , Yan, Z. B. , Niklas, K. J. , Han, W. X. , Kattge, J. , Reich, P. B. , Luo, Y. , Chen, Y. , Tang, Z. , Hu, H. , Wright, I. J. , Schmid, B. , & Fang, J. Y. (2018). Global leaf nitrogen and phosphorus stoichiometry and their scaling exponent. National Science Review, 5(5), 728–739. 10.1093/nsr/nwx142

[ece370152-bib-0043] Townsend, A. R. , Cleveland, C. C. , Asner, G. P. , & Bustamante, M. M. C. (2007). Controls over foliar N:P ratios in tropical rain forests. Ecology, 88(1), 107–118. 10.1890/0012-9658(2007)88[107:Cofnri]2.0.Co;2 17489459

[ece370152-bib-0044] Wang, L. J. , Zhao, M. L. , Wei, S. Y. , Song, W. M. , Chu, X. J. , Li, P. G. , Wang, X. , Zhang, X. , Cao, Q. , & Han, G. X. (2024). Inundation depth controls leaf photosynthetic capacity by regulating leaf area and N content in an estuarine wetland. Plant and Soil, 496(1–2), 375–390. 10.1007/s11104-023-06368-x

[ece370152-bib-0045] Wu, T. G. , Yu, M. K. , Wang, G. G. , Dong, Y. , & Cheng, X. R. (2012). Leaf nitrogen and phosphorus stoichiometry across forty‐two woody species in Southeast China. Biochemical Systematics and Ecology, 44, 255–263. 10.1016/j.bse.2012.06.002

[ece370152-bib-0046] Xing, K. X. , Niinemets, Ü. , Rengel, Z. , Onoda, Y. , Xia, J. Z. , Chen, H. Y. H. , Zhao, M. , Han, W. , & Li, H. B. (2021). Global patterns of leaf construction traits and their covariation along climate and soil environmental gradients. New Phytologist, 232(4), 1648–1660. 10.1111/nph.17686 34418102

[ece370152-bib-0047] Yan, B. , Yang, X. , Zhou, L. F. , & Wang, C. (2016). Seasonal variations and trend prediction of upstream water quality of Dahuofang reservoir in Hunhe River. Toxicological and Environmental Chemistry, 98(3–4), 345–357. 10.1080/02772248.2015.1123479

[ece370152-bib-0048] Yan, Z. B. , Kim, N. , Han, W. X. , Guo, Y. L. , Han, T. S. , Du, E. Z. , & Fang, J. Y. (2015). Effects of nitrogen and phosphorus supply on growth rate, leaf stoichiometry, and nutrient resorption of *Arabidopsis thaliana* . Plant and Soil, 388(1–2), 147–155. 10.1007/s11104-014-2316-1

[ece370152-bib-0049] Yang, G. , Li, Y. , Huang, T. Q. , Fu, B. L. , Tang, J. , Zhang, X. , & Wu, J. S. (2021). Multi‐scale evaluation of ecological restoration effects in the riparian zone using Landsat series images from 1980 to 2019. Ecological Indicators, 132, 108342. 10.1016/j.ecolind.2021.108342

[ece370152-bib-0050] Yu, M. F. , Tao, Y. X. , Liu, W. Z. , Xing, W. , Liu, G. H. , Wang, L. , & Ma, L. (2020). C, N, and P stoichiometry and their interaction with different plant communities and soils in subtropical riparian wetlands. Environmental Science and Pollution Research, 27(1), 1024–1034. 10.1007/s11356-019-07004-x 31820250

[ece370152-bib-0051] Yu, Q. , Chen, Q. S. , Elser, J. J. , He, N. P. , Wu, H. H. , Zhang, G. M. , Wu, J. , Bai, Y. , & Han, X. G. (2010). Linking stoichiometric homoeostasis with ecosystem structure, functioning and stability. Ecology Letters, 13(11), 1390–1399. 10.1111/j.1461-0248.2010.01532.x 20849443

[ece370152-bib-0052] Zhang, H. , Yang, X. Q. , Wang, J. Y. , Wang, G. G. , Yu, M. K. , & Wu, T. G. (2017). Leaf N and P stoichiometry in relation to leaf shape and plant size for Quercus acutissima provenances across China. Scientific Reports, 7, 46133. 10.1038/srep46133 28393848 PMC5385868

[ece370152-bib-0053] Zhang, J. H. , Li, M. X. , Xu, L. , Zhu, J. X. , Dai, G. H. , & He, N. P. (2021). C:N:P stoichiometry in terrestrial ecosystems in China. Science of the Total Environment, 795, 8849. 10.1016/j.scitotenv.2021.148849 34246133

[ece370152-bib-0054] Zhang, J. H. , Zhao, N. , Liu, C. C. , Yang, H. , Li, M. L. , Yu, G. R. , & He, N. P. (2018). C:N:P stoichiometry in China's forests: From organs to ecosystems. Functional Ecology, 32(1), 50–60. 10.1111/1365-2435.12979

[ece370152-bib-0055] Zhang, P. , Wang, H. , Wu, Q. T. , Yu, M. K. , & Wu, T. G. (2018). Effect of wind on the relation of leaf N, P stoichiometry with leaf morphology in Quercus species. Forests, 9(3), 30110. 10.3390/f9030110

